# Text-to-hierarchical three-dimensional scene generation: a new approach for layered three-dimensional modeling from natural language

**DOI:** 10.1186/s42492-026-00226-0

**Published:** 2026-08-26

**Authors:** Zuan Gu, Tianhan Gao, Lanxu Zhao, Huimin Liu

**Affiliations:** Northeastern University, Shenyang, Liaoning 110167 China

**Keywords:** Text-to-3D scene generation, Hierarchical modeling, 3D Gaussian splatting, Scene-object decoupling

## Abstract

Rapid advancements in generative models have enabled substantial progress in text-to-3D scene synthesis. However, existing approaches often lack hierarchical structure and geometric consistency, limiting their use in complex, editable scene creation. To address this issue, an end-to-end hierarchical framework for text-to-3D scene generation is introduced. The main contribution of this study lies not in advancing a single generative model but in the design of a framework that synergistically integrates state-of-the-art components for video synthesis and mesh reconstruction. By decoupling scene semantics from dynamic camera trajectory control, the proposed system efficiently produces structured object-level editable three-dimensional scenes from natural language. Comprehensive experiments showed that the proposed integrated approach achieved superior scene quality, consistency, and editing flexibility compared with existing methods, offering a powerful solution for applications, such as virtual reality and digital twins.

## Introduction

Recent breakthroughs in generative artificial intelligence are transforming three-dimensional (3D) content creation, particularly text-driven scene generation, with applications ranging from virtual reality to digital twins. Although pioneering methods, such as DreamFusion [1] and Shap-E [2], achieve quality single-object synthesis through neural radiance fields (NeRFs) and diffusion model integration, they face fundamental limitations in complex scene modeling [3–5]. Traditional monolithic end-to-end generation paradigms produce tightly coupled scene elements at the geometric-semantic level, critically limiting model editability and physical plausibility. These constraints manifest prominently in three aspects: static synthesis prevents dynamic camera control, sparse-view reconstruction yields noisy geometries, and fused scene meshes prohibit component-level editing, thereby revealing the core bottleneck of inadequate hierarchical structural modeling and dynamic interaction capabilities. Recent *Visual Computing for Industry, Biomedicine, and Art* (VCIBA) studies have highlighted geometry-aware representations and efficient rendering priors that strengthen 3D consistency, e.g., precise 3D reconstruction from single images [6], real-time volume rendering optimizations [7], and high-fidelity text-to-3D scene generation frameworks that integrate three-dimensional Gaussian splatting (3DGS) [8].

To address this challenge, a hierarchical 3D scene generation method that achieves the precise mapping of natural language descriptions to structured, editable 3D scenes is proposed. The primary contribution of this study is not advancing individual generative models but designing an end-to-end hierarchical framework that synergistically combines state-of-the-art text-to-video synthesis with robust mesh reconstruction techniques. Although an established methodology for object generation inspired by TRELLIS [9] is leveraged, the innovation of this study lies in the complete pipeline that uniquely enables dynamic trajectory control and high-degree scene editability. In addition, recent VCIBA reports on structure-aware 3D reconstructions from sparse or single views [6] have reinforced the coarse-to-fine design choices in this study for geometry preservation under limited observations.

To properly situate this work, it is crucial to distinguish between the two dominant paradigms in structured 3D scene creation: (1) single-view reconstruction, which aims to infer a 3D scene from preexisting two-dimensional (2D) pixel data, and (2) text-conditioned generation, which aims to synthesize a 3D scene from an abstract linguistic description. This study belongs to the second category [6, 10].

Second, rather than proposing a fundamentally new segmentation architecture, an effective system-level integration that adapts to existing visual foundation models (e.g., segment anything model (SAM)) and cross-modal attention mechanisms is proposed. Through this task-specific integration, a robust separation of target objects from backgrounds in video streams was achieved. Building on this, the proposed system employed dual 3D representation: sparse 3DGS was utilized to optimize the scene background, whereas structured latent representation models drove the independent generation of explicit meshes for foreground objects [7, 11, 12]. The motivation behind this hybrid representation is twofold. While pure 3DGS offers superior rendering efficiency and visual fidelity for complex, large-scale backgrounds, it inherently hinders fine-grained, physics-aware object manipulation (e.g., collision detection and rigid-body transformations in engines, such as Unity). Conversely, applying a purely mesh-based approach to an entire scene is computationally prohibitive and prone to geometric artifacts. By decoupling these two modalities, the proposed framework effectively bridges the gap between the rendering efficiency and physical editability. Practically, modeling an environment with 3DGS aligns with the IVC evidence of its efficiency and editability [13], whereas instance-level separation can draw on retrieval-driven priors to stabilize object reconstruction [14]. Finally, hierarchical composition was implemented through geometry-projection-constrained object localization. Practically, modeling an environment with 3DGS aligns with recent VCIBA evidence of its real-time rendering efficiency and scene editability [8].

This study makes the following contributions.

- **Independent trajectory-text decoupling**: Parametric three-dimensional special Euclidean group SE(3) camera motion control combined with spatiotemporal diffusion modeling enables the separate regulation of text semantics and camera trajectories, generating multiview sequences with geometric consistency for downstream processing.
- **System-level hierarchical integration for scene generation**: A comprehensive framework that synergistically integrates state-of-the-art components for video synthesis, segmentation (e.g., SAM), and 3D reconstruction is introduced. It effectively separates objects from the background in generated video sequences. By leveraging distinct, optimized 3D representations for the environment (via 3DGS) and individual objects (via an adapted mesh generation model), the integrated system overcomes the limitations of monolithic representations, enabling vertex-level editing and physically consistent scene integration [13–15].

The experimental results demonstrate the effectiveness of the proposed method in complex indoor scenes (e.g., art galleries and industrial lofts), successfully decomposing scenes while enabling real-time interactive editing. These advances have established new technical paradigms for dynamic scene generation and digital twin applications.

### Text-to-3D generation

Recent text-to-3D methods have used diffusion models, NeRF[16], and 3DGS. As highlighted in recent comprehensive reviews of generative 3D reconstruction [17], this field is rapidly evolving with novel approaches even leveraging continuous normalizing flows for point-cloud generation [18]. DreamFusion uses score distillation sampling to optimize NeRF for text-driven, category-agnostic 3D synthesis [1, 19, 20]. Shap-E and GSGEN combine vision–language models with 3DGS to stabilize text–geometry alignment. Diffusion models generate diverse 3D scenes from text and support real-time stylization (e.g., X-Mesh [21]). Text-to-video frameworks, such as OpenSora [22] and Stable Video Diffusion [23], enable camera-aware videos from text. New approaches (e.g., CamCo [24], ViewCrafter[25], VD3D [26]) use transformer-based 3D diffusion to enhance geometric consistency.

Early scene-level generative models typically employed a monolithic paradigm (e.g., DreamFusion, Text2Room, Text2NeRF), weaving all the scene elements into a single representation. Although effective for static visual synthesis, they often suffer from geometric-semantic entanglement, which severely limits object-level editability. To mitigate this, recent structure-aware or compositional text-to-3D frameworks, such as Set-the-Scene [27], GALA3D [28], and CG3D [29], have emerged. These methods use cross-modal conditioning and instance-level layout generation to achieve compositional control. However, these methods typically rely on explicitly provided 3D bounding boxes, spatial layouts, or scene graphs as prior condition inputs. In contrast, the proposed pipeline derives an object-background decoupled representation directly from natural language and dynamic camera trajectories, uniquely addressing continuous multiview background extraction without requiring predefined explicit 3D layouts.

In addition to reconstructing the overall scene, the proposed hierarchical approach requires the robust generation of individual object meshes from masked image sequences. Several effective methods have been developed for this purpose. TRELLIS [9] introduced a robust framework that decouples geometry and appearance encoding to generate high-fidelity meshes from sparse views. Given its proven effectiveness, a TRELLIS-like architecture was adopted for the object-generation module. Consequently, this study focuses on seamlessly integrating this powerful object-level generator into a larger scene-level framework driven by dynamic camera trajectories, which is a nontrivial challenge that existing methods have not addressed.

### Image sequence interaction

New frameworks (e.g., SAM and SAM2 [30, 31]) enable real-time interactive segmentation with high accuracy, surpassing traditional methods, such as MOTS [32], OSTrack [33], and YOLO [34]. Integration with auto-annotation tools, such as Florence [35], improves scalability. Combined with advanced inpainting and synthesis tools (e.g., LaMa [36] and LDM [37]), this supports object-level editing in videos and enhances the reliability and flexibility of dynamic scene understanding.

### Sparse 3D scene reconstruction

**Fig. 1:**
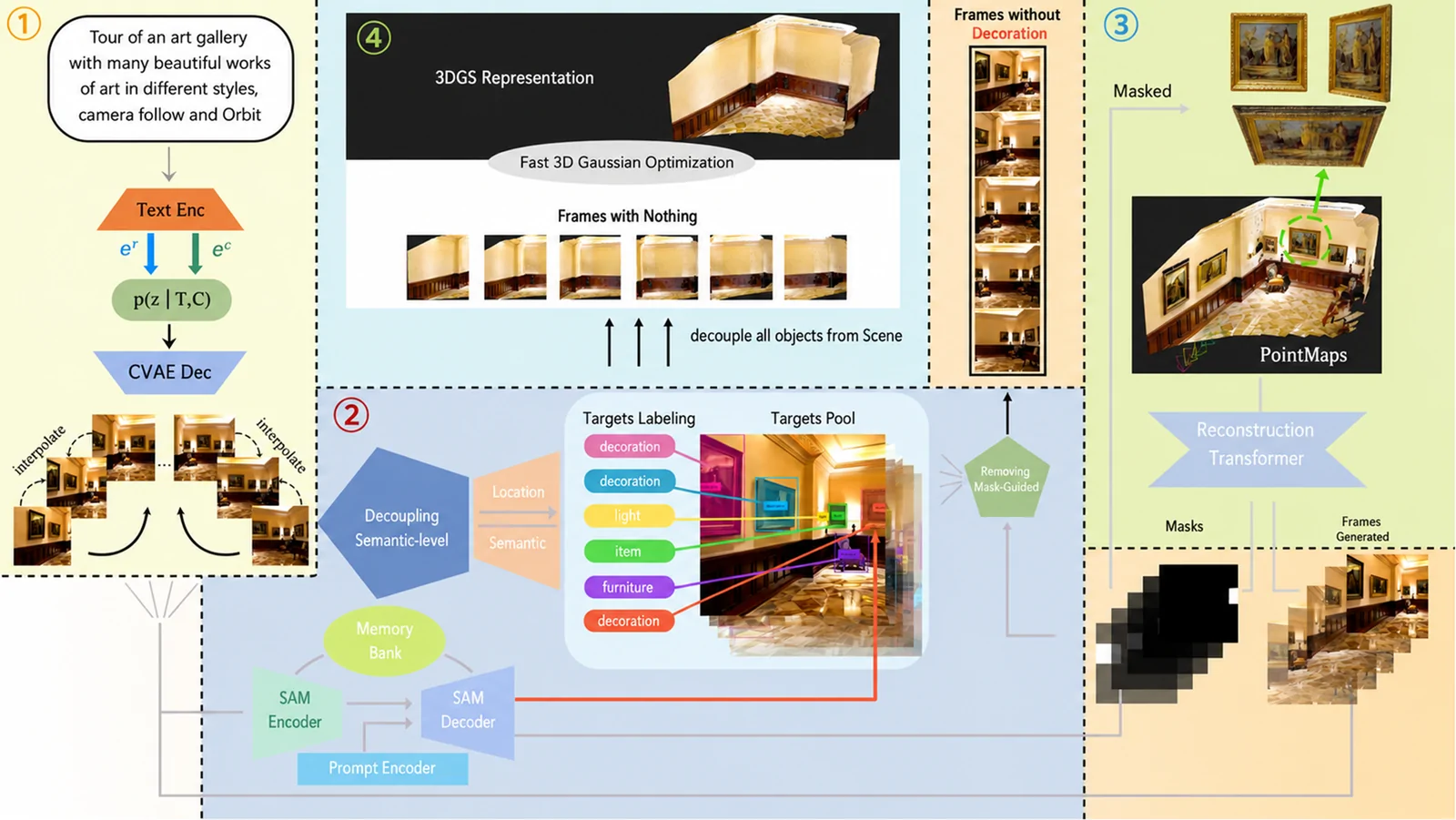
Overview of the compositional text-to-3D scene pipeline. (1) Given a free-form textual description, a conditional variational autoencoder (CVAE)-based video generator produces a sequence of plausible camera-follow frames. (2) Semantic decoupling is performed on these frames with segment anything model (SAM) and a memory bank, progressively separating foreground targets (e.g., decorations, lights, furniture) from the background and building a labeled target pool while obtaining clean, object-removed background frames. (3) Foreground objects are reconstructed using a Mast3R-style point-map module and a reconstruction transformer under mask guidance, yielding view-consistent two-dimensional assets and masks. (4) The background is reconstructed via fast three-dimensional Gaussian splatting (3DGS) from the cleaned frames (Mast3R + 3DGS), and the final three-dimensional (3D) scene mesh is obtained by composing foreground assets into the 3D background according to estimated object poses and camera trajectory

Sparse-view scene reconstruction aims at recovering 3D structures from a limited visual perspective. Existing approaches can be categorized into two paradigms: (1) Cross-scene generalization methods [38–40] that learn scene-agnostic priors for novel view synthesis, and (2) Per-scene optimization strategies [41, 42] incorporating regularization mechanisms for instance-specific refinement. The emergence of Gaussian splatting has propelled advancements in this domain. FSGS [43] enhances sparse view reconstruction through proximity-guided Gaussian decomposition and pretrained 2D depth estimation networks to improve depth supervision during rendering. InstantSplat [44] achieves rapid reconstruction by leveraging DUSt3R [45] to predict initial 3D point maps and camera parameters from ultrasparse inputs, followed by joint optimization with pose regularization constraints for geometric consistency. Figure 1 summarizes the full pipeline from text-conditioned video generation to hierarchical scene composition.

### Reconstructing 3D scenes from a single image

A distinct but related line of research focused on reconstructing a 3D scene from a single red, green, and blue image. Methods, such as CAST[46], operate within this reconstruction paradigm. The workflow for such systems begins by analyzing a single input image to extract object-level 2D segmentations and estimate the relative depth. Subsequent steps often involve using large language models to infer the spatial relationships between objects and applying physics-aware correction mechanisms to ensure that the final reconstructed scene is coherent and plausible. Although these reconstruction methods are powerful for interpreting existing visual data, this study addresses the distinct challenge of scene synthesis from language, which necessitates a fundamentally different approach rooted in generative modeling rather than geometric inference.

## Methods

### Text-to-image sequence with trajectory control

**Fig. 2:**
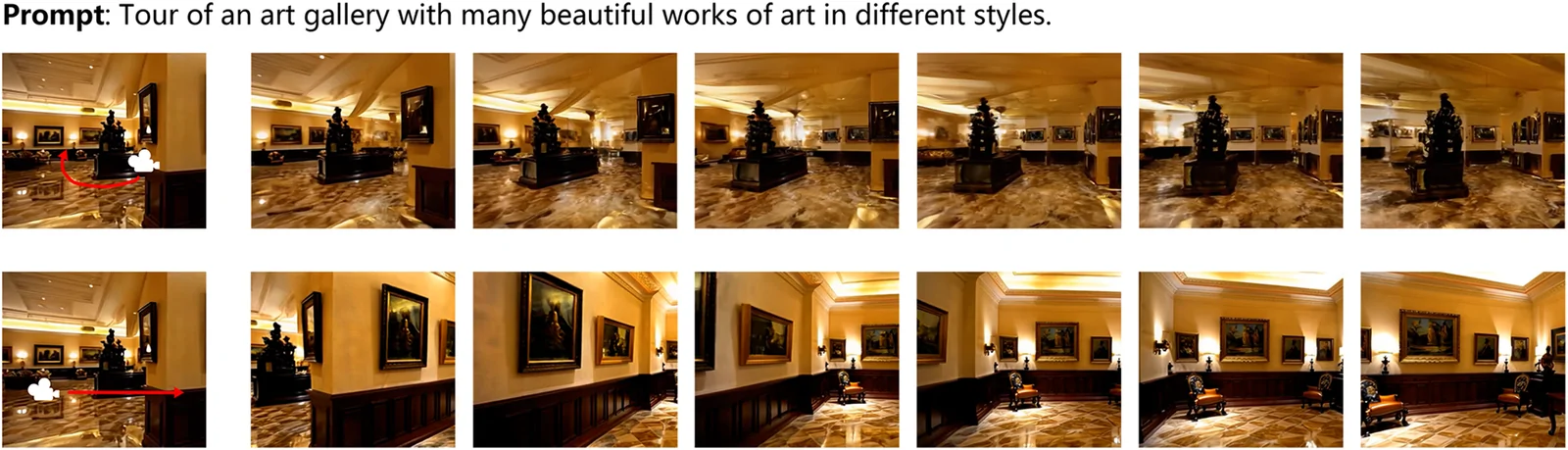
Generative scene modeling process, demonstrating that the prescribed camera trajectories effectively constrained by the original frames achieve consistent content generation

As illustrated in Fig. 2, given a natural-language text prompt (input), this stage produces a sequence of images with continuous content and strong geometric consistency across views (output). A conditional variational autoencoder (CVAE) conditioned on the camera trajectory with an encoder $q_\phi(z \mid x, c)$ and a decoder $p_\theta(x \mid z, c)$ that map between images *x*, latents *z*, and camera poses *c* is used. By decoupling scene semantics from camera motion, this framework ensures that images from different viewpoints remain coherent and structurally complete, avoiding the ‘holes’ often seen in multiview generation.

The camera motion was explicitly parameterized on a continuous SE(3) manifold. Specifically, the trajectory is modeled as

1 $$C(t) = \left( \exp(\omega(t)^\wedge),\ t(t) \right) \in \mathrm{SE}(3)$$

where $\omega(t) \in \mathfrak{so}(3)$ is the rotation axis, and *t(t)* is the translation (modeled via cubic B-spline interpolation). These trajectory parameters are then encoded into latent code $e_c = P_\psi([\omega(t); t(t)])$ using network $P_\psi$. This yields a trajectory-conditioned latent embedding for the generative model.

To fuse text and trajectory, a spatiotemporally decoupled diffusion process is used. In the forward process, Gaussian noise is gradually added to the latent variables.

2 $$q(v_{1:T} \mid v_0) = \prod_{t=1}^{T} \mathcal{N}(v_t; \sqrt{\alpha_t} v_{t-1},\ (1 - \alpha_t) I)$$

The reverse denoising network has two branches. The spatial branch applies 3D convolutions to extract local visual features (denoted $\epsilon_{\mathrm{spa},\theta} = \texttt{Conv3D}(v_t)$), which are fused with text embeddings. The temporal branch incorporates the camera trajectory via constrained interpolation $\varphi(\Delta C) = \sum_{k=0}^{K} \beta_k B_k(t)$ (with cubic B-spline basis functions $B_k(t)$), where the coefficients $\beta_k$ depend on trajectory changes. By combining explicit $\mathrm{SE}(3)$-trajectory parameterization and a gated attention mechanism in this dual-path design, the model achieves the independent control of camera motion and scene semantics during generation.

The CVAE is trained using the composite loss:

3 $$\eqalign{ L & = {\lambda _E}{{\cal L}_E} + {\lambda _{{\rm{align}}}}{{\cal L}_{{\rm{align}}}} \cr & + {\lambda _{{\rm{smooth}}}}{{\cal L}_{{\rm{smooth}}}} + {\lambda _{{\rm{sparse}}}}{{\cal L}_{{\rm{sparse}}}}\cr} $$

Here, $\mathcal{L}_E$ is the evidence lower bound (negative log-likelihood plus weighted Kullback-Leibler (KL) divergence). $\mathcal{L}_{\mathrm{align}}$ is the geometric alignment loss that enforces consistency between the camera pose inferred from the generated keyframes and the specified trajectory. $\mathcal{L}_{\mathrm{smooth}}$ penalizes high accelerations ($\partial^2 C(t)/\partial t^2$) to encourage smooth motion, and $\mathcal{L}_{\mathrm{sparse}}$ is a sparsity regularizer (including an $L_1$-norm on *z*) that promotes a compact latent representation and prevents unnecessary changes between frames.

At the time of inference, the joint sampling of the semantic and trajectory-conditioned latents was performed. *z* was drawn from the prior *p(z ∣ T, C)* conditioned on text *T* and the camera trajectory *C*. The decoder then generated the base keyframe images at the selected trajectory times $t_k$:

4 $$I_{\mathrm{base}}^{(k)} = D_\theta(z, C(t_k))$$

These base frames served as anchors for constructing a full multiview image sequence.

### Image sequence to hierarchical scene model generation

This stage converts the multiview image sequence (subsection “Quantitative results”) into a hierarchical 3D scene representation using a reconstructed background mesh and object meshes for each foreground instance. The foreground objects are isolated and removed, yielding object-specific image sets and a clean background sequence. The background sequence is reconstructed using a sparse Gaussian representation, and each foreground object is reconstructed into a detailed 3D mesh. These components form a layered scene model with a distinct background and movable objects.

#### Multi-object hierarchy construction

First, the multiview image sequence $I=\{I_t\}_{t=1}^T$ is processed to separate each object from the scene and background. A hierarchical visual encoder extracts multiscale spatiotemporal features, whereas a text encoder maps category labels to semantic embeddings. A gated cross-modal attention mechanism then disentangles the object features from the background context. The initial object candidates (bounding box $B^i_1$, binary mask $M^i_{1,\mathrm{init}}$, and label $c_i$) are generated in the first frame according to the predicted categories.

**Fig. 3:**
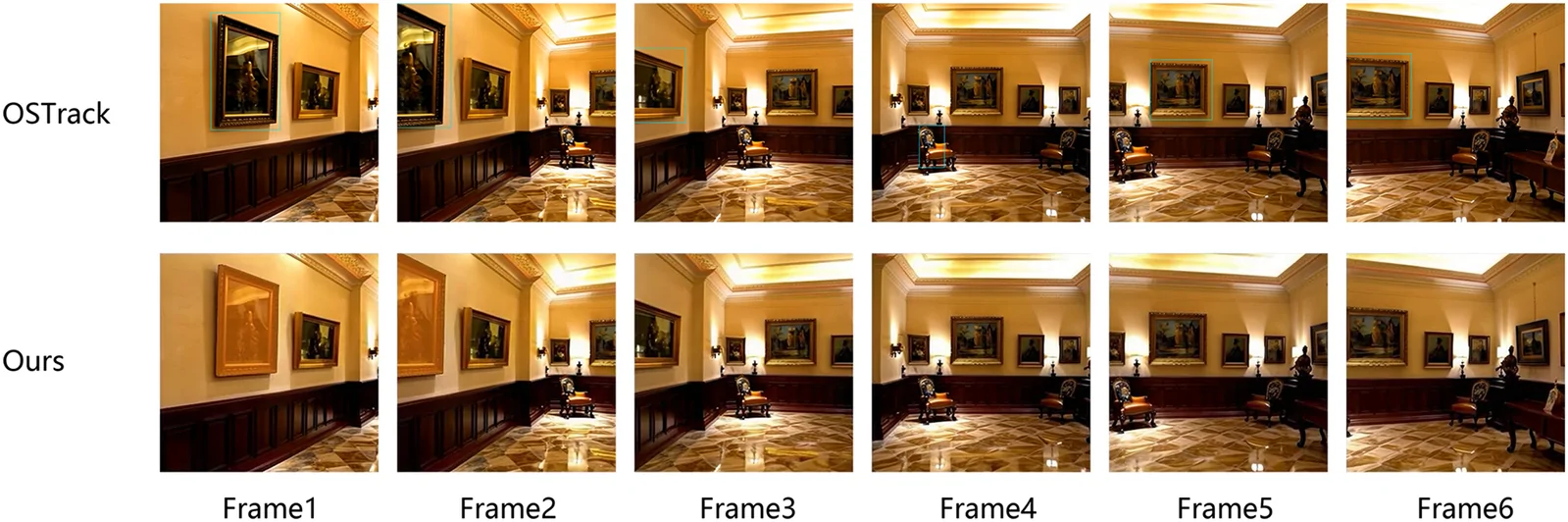
Consistent tracking for the primary target (‘artwork’) achieved using the proposed method. Unlike OSTrack [34], which fails when targets exit the frame, the proposed approach overcomes this, demonstrating enhanced robustness

Dynamic instance tracking is then performed to propagate each object mask $M^i_t$ over time: For object $o_i$, features are pooled from the previous frame’s region of interest and fused with the memory of past features (managed as a target pool) to refine the mask. To address the challenge of severe occlusions in long video sequences, the target pool acts as a temporal memory bank. It dynamically aggregates object features from highly confident unoccluded frames. When an object is heavily obscured, the system retrieves these historical features to maintain temporal and semantic continuity, thereby preventing tracking losses. A cascaded segmentation head iteratively updates $M^i_t$ and refines bounding box $B^i_t$. After tracking, the images of each object are extracted by pixel-wise masking:

5 $$O_t^i = I_t \odot M_t^i$$

which yields an object-specific image sequence that isolates $o_i$ from the background. Figure 3 shows that the memory-assisted tracking remains stable when the target leaves and reenters the frame.

**Fig. 4:**
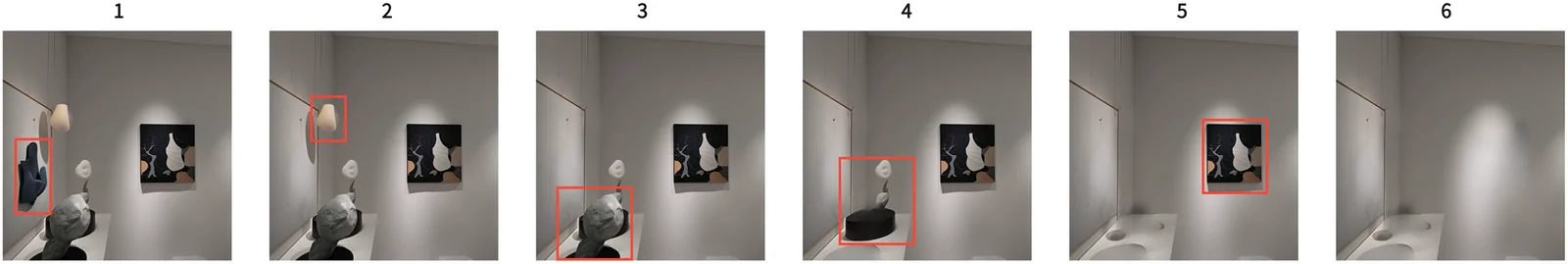
The figure midas-guided [47] sequential inpainting using left-right/front-back order. Stages 3–4 successfully reconstruct occluded objects, validating the scene restoration capabilities of the proposed method

Finally, the background is restored using latent-space inpainting. A multi-object erasure mask $\Omega_t=\bigcup_i M^i_t$ is formed to mark all the foreground regions. In the latent space $z_t = E(I_t)$, the missing background is filled by injecting noise into the erased regions and decoding

6 $$z_t^{\mathrm{clean}} = D_\theta\left(z_t \odot (1 - \Omega_t) + \epsilon \odot \Omega_t\right), \quad \epsilon \sim \mathcal{N}(0, \mathbf{I})$$

to the inpainted image $\tilde{I}_t=G(z^{\mathrm{clean}}_t)$. This yields a complete background sequence with plausibly filled occluded areas, as shown in Fig. 4.

#### 3D reconstruction of background image sequences

Inspired by classical multiview stereo (MVS), coarse camera poses and a sparse point cloud are first recovered for the background scene. In particular, the MASt3R foundation model is leveraged to produce initial extrinsics ${T_v}$ and pixel-aligned 3D points ${P_v}$ from multiview image correspondences. Sparse keypoints across the image sequence are detected and matched, and then triangulated to seed the initial reconstruction. For each image pair $(I_i, I_j)$, the relative pose and per-frame depth that minimize the feature reprojection error are determined, effectively triangulating the matched pixels. For robustness, random sample consensus is applied to reject outliers in each pairwise pose estimation. A global pose graph is constructed and a bundle adjustment is performed to enforce consistency and fix the scale across all the views. The result is a set of refined camera extrinsics and a dense sparse point cloud covering the background.

Building on the refined points, a 2D Gaussian splatting representation of the scene is adopted. Each 3D point *i* is represented as an anisotropic Gaussian with mean $\mu_i$ and covariance $\Sigma_i$. Under camera *v*, this Gaussian projects onto the image plane as an ellipse; its mean projects to $u = \pi(T_v \mu_i)$, and its 2D covariance becomes $\Sigma^{\prime}_i$. The intensity contribution at each pixel *x* is defined using the following Gaussian formula:

7 $$G_i(x) = \exp\left( -\frac{1}{2} (x - u)^\top \Sigma_i^{\prime -1} (x - u) \right)$$

where *x* denotes the pixel coordinates. Summing these contributions for all Gaussians yields a continuous background radiance field. In practice, each Gaussian also carries a weight or color (e.g., initialized from image intensities). However, this study focuses on geometric occupancy. This compact model produces a smooth density field over the scene volume, ensuring that even sparse points contribute to a continuous effect for each view.

#### 3D object mesh generation via an adapted framework

**Fig. 5:**
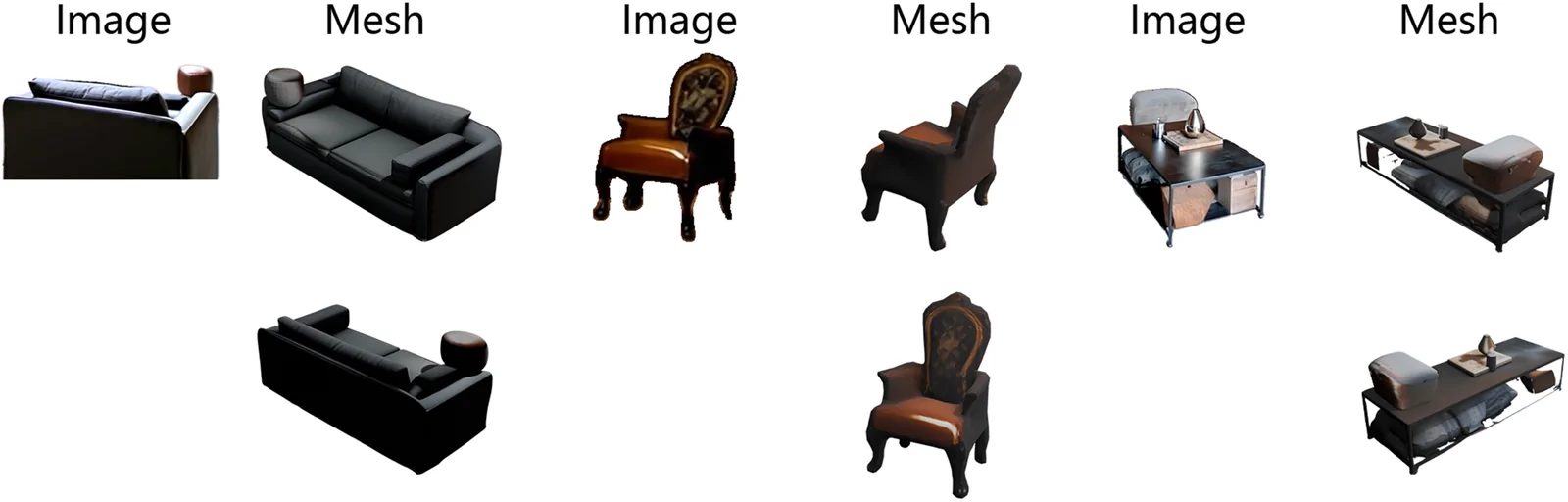
Reconstruction of three-dimensional geometry from temporally ordered multiview image sequences using the proposed framework

As illustrated in Fig. 5, to generate a 3D mesh for each target object $o_i$, the successful TRELLIS framework [9] was utilized. The process begins by taking masked multiview images $O_t$ along with the object’s text prompt $c_i$ as input. In this joint generation process, the 2D masks (which isolate $O_t$) primarily provide the geometric bounds and spatial localization. The text prompt acts as a semantic prior. By leveraging the strong generative priors of the pretrained model, the text prompt supplements and infers texture and geometric details for regions that are heavily occluded or imperfectly segmented by SAM, thereby reducing the sensitivity of the final geometry to segmentation artifacts. Following the core principles of TRELLIS, the global geometry is decoupled from the local appearance by employing a dual-encoder architecture to separately extract the geometric and appearance features. The per-view features are fused into latent codes via temporal attention; specifically, the latent means are computed by

8 $$\mu_k = \sum_{t=1}^{T} \alpha_t E_k(O_t) \quad $$

9 $$\alpha_t = \mathrm{softmax} \left( W_k \begin{bmatrix} E_g(O_t); E_a(O_t) \end{bmatrix} \right), \quad k \in \{g, a\}$$

where $W_k$ denotes learned weights. This yields the aggregated Gaussian parameters $(\mu_g,\Sigma_g)$ and $(\mu_a,\Sigma_a)$ that define the distributions of $z_g$ and $z_a$.

In the decoding stage, the geometry decoder $D_g$ takes $z_g$, predicts an implicit signed distance field (SDF), $SDF(x)=D_g(x,z_g)$, and extracts the object mesh by applying differentiable marching cubes to the zero-level set. The appearance decoder $D_a$ takes $z_a$ and predicts the per-vertex texture coordinates $U_i$ and the material parameters $\rho_i$. A differentiable renderer *R* enforces multiview consistency by projecting a textured mesh onto each camera view. The model was trained end-to-end with losses in the image reconstruction, latent (KL) regularization, and geometry smoothness (Eikonal), yielding a final textured mesh for each object.

Finally, an explicit mesh was extracted by converting the Gaussian field into a volumetric representation. The Gaussians were rasterized into a 3D occupancy grid, and Poisson surface reconstruction was applied to obtain a smooth, watertight triangular mesh. The density was thresholded to remove the isolated components and noise. The final mesh captured the background geometry with fine details and a smooth topology.

### Hierarchical scene integration

The inputs to this stage include the multiview image sequence, object masks from the separation stage, reconstructed object meshes, and background mesh. The output is an integrated scene model with all objects correctly placed in the background. To align the reconstructed scene with the original observations, a geometric correspondence is established using a projection-based pipeline. Specifically, the initial position of each object is computed by backprojecting the center of its 2D bounding box $(u_i, v_i)$ from a reference view into 3D using the depth map $D_{\mathrm{ref}}$:

10 $$t_i^{(0)} = T_v^{-1} \left( d_i K^{-1} \begin{bmatrix} u_i, v_i, 1 \end{bmatrix}^\top \right)$$

where $T_v$ is the camera extrinsic matrix, and $d_i = D_{\mathrm{ref}}(u_i, v_i)$. This anchors each object at the intersection of the camera ray through $(u_i, v_i)$ and the estimated depth to ensure an initial alignment with the observations.

The initial scale is estimated by matching the median depth of the object mask with the median size of the object model. Let $\Omega_i$ be the pixel set of the object mask, and let $p_i^O$ be the coordinates of the object model. Then,

11 $$s_i^{(0)} = \frac{\mathrm{median}\left(D_{\mathrm{ref}}(\Omega_i)\right)}{\mathrm{median}\left(\left\|p_i^O\right\|_2\right)}$$

where $\mathrm{median}(D_{\mathrm{ref}}(\Omega_i))$ is the median depth of the mask region, and $\mathrm{median}(|p_i^O|_2)$ is the median distance of the model vertices from its center. The initial rotation is set by aligning the object’s principal axis with the dominant normal in the scene as follows:

12 $$R_i^{(0)} = \mathrm{Align}(v_1, n_{\mathrm{scene}})s$$

where $v_1$ is the first principal direction of the object model (from principal component analysis), and $n_{\mathrm{scene}}$ is the dominant surface normal in the scene.

Finally, the object parameters $\Theta_i = {R_i, s_i, t_i}$ are refined by minimizing the composite objective as follows:

13 $$\Theta_i^* = \arg\min_{\Theta_i} \sum_{v=1}^{N} \mathcal{L}_{\mathrm{align}} + \lambda \mathcal{L}_{\mathrm{photo}} + \mu \mathcal{L}_{\mathrm{coll}}$$

Here, the geometric alignment term $L_{\mathrm{align}}$ enforces consistency between the projected object surface and the observed 3D structure (e.g., by minimizing distances to the multiview point clouds), the photometric term $L_{\mathrm{photo}}$ enforces the color and silhouette consistency of the object across views, and the collision term $L_{\mathrm{coll}}$ penalizes interobject penetration (for example via a SDF). Together, these terms ensure that objects are positioned, scaled, and oriented to best fit the image evidence while remaining collision-free.

### Datasets and baselines

#### Datasets

To ensure a fair and reproducible evaluation of the generalization capabilities of the proposed method, experiments were conducted on two widely used standard datasets. This study utilized Hypersim [48], a dataset of high-quality indoor scenes featuring complex physical lighting and multi-object interactions, making it ideal for assessing rendering quality and geometric detail. ScanNet [49], a large-scale dataset of real-world scanned environments, was also employed, and it served as a crucial benchmark for validating the robustness of the proposed method to real-world data. In-house synthetic dataset (Unity 25), in addition to public benchmarks, a virtual dataset in Unity comprising 25 scene models spanning four categories, namely, industrial, home, fantasy, and sci-fi, was constructed. Each scene was authored with physically based rendering materials and physically plausible illumination using high dynamic range imaging and emissive sources, and rendered under consistent camera intrinsics to facilitate cross-scene comparisons. For every scene, multiview image sequences accompanied by camera poses on SE(3), depth maps, and instance masks were exported. Where applicable, part-level labels were also provided for object-centric analyses. The dataset is intended for research use; metadata include scene category, prompt text, trajectory type, and rendering settings to enable the controlled studies of hierarchy, trajectory control, and reconstruction fidelity.

#### Baselines

To situate the contributions of this study, the quantitative evaluations focused on three baseline models that epitomized layout-free, end-to-end paradigms in text-to-3D scene generation. Specifically, the following were selected: (1) Text2Room [5], a pioneering method that combines procedural and generative techniques but jointly optimizes geometry and appearance; (2) Text2NeRF [4], which is representative of the monolithic neural field paradigm, optimizing a single unified NeRF for the entire scene; and (3) DreamScene [3], which introduces 3DGS for significantly improved rendering efficiency but still treats the scene as a single, holistic entity for optimization.

Note that, while recent compositional methods (e.g., set-the-scene [27] and GALA3D [28]) have emerged, they fundamentally differ from the problem setting in this study by requiring predefined explicit 3D layouts. A detailed capability comparison and rationale for the baseline selection in this study are discussed at the beginning of the Results and Discussion section.

### Quantitative metrics

To objectively assess the quality of the generated scenes, a comprehensive set of standard quantitative metrics spanning both 2D rendering quality and 3D geometric fidelity was employed.

**Image-based rendering quality.** The quality of the rendered novel views was evaluated from multiple perspectives. Herein, the peak signal-to-noise ratio (PSNR) for pixel-level fidelity is reported. As the primary metric, learned perceptual image patch similarity (LPIPS), which leverages deep features to better approximate the human perceptual judgment of realism, was used.

**3D geometric fidelity.** The geometric accuracy was quantified by comparing the generated models with their ground-truth counterparts. Chamfer distance (CD) was used to measure the overall shape alignment. Furthermore, to precisely evaluate the geometry of foreground objects, the Earth mover’s distance (EMD), which is more sensitive to fine-grained details and topology, was employed.

### Implementation details

**Video generation module.** Built upon Stable Video Diffusion XL3 [23], trajectory parameters were sampled at intervals of 0.1 s to generate 1024 *×* 1024 resolution sequences. This resolution balances the generation efficiency with multiscale detail preservation (Fig. 2 for the trajectory-conditioned generation. For the CVAE-based architecture, the network was trained using the AdamW optimizer ($\beta_1=0.9, \beta_2=0.999$) with an initial learning rate of $1 \times 10^{-4}$ and a cosine annealing schedule. The training process required 150 epochs, with a batch size of 16.

**Object mesh generation.** For the object generation module built on TRELLIS, the majority of the pretrained backbone was frozen to retain its strong structural priors. Only the latent adapter layers for 50 epochs were finetuned using a reduced learning rate of $5 \times 10^{-5}.$ This targeted adaptation ensured stable convergence while successfully aligning the generated meshes with the extracted 2D video masks.

**3D scene reconstruction.** Here, 30K iterations for 3DGS were conducted by employing dynamic Gaussian pruning to compress the geometry. After every 5K iterations, low-contribution Gaussians were filtered based on the impact scores, achieving a 68% storage reduction (from 120K to 72K) without quality degradation (LPIPS difference * < 0.02*).

**Datasets and evaluation protocol.** The framework was evaluated across three datasets. Hypersim was partitioned into 360/45/45 (train/val/test) scenes, and ScanNet was partitioned into 80/10/10 scenes with clear object boundaries. The custom Unity-25 dataset was used exclusively for the zero-shot generalization testing. For a comprehensive generative evaluation, a testing suite comprising 30 representative text prompts spanning three distinct scenarios, namely, industrial manufacturing (to assess complex geometric structures), art galleries (for lighting and material variations), and domestic living spaces (to evaluate object-dense layouts), was curated. Each prompt was formulated to contain at least two distinct foreground instances to explicitly validate the proposed hierarchical decoupling mechanism. Furthermore, to objectively and rigorously evaluate the accuracy of 3D reconstruction metrics (e.g., PSNR, structural similarity index measure, LPIPS), a conditional testing variant was introduced in which the textual prompts were replaced with sparse images sampled from the test set as condition inputs. This enabled the isolation of the evaluation focus and the benchmarking of the pipeline directly from the reconstruction and mesh generation stages.

**Hardware configuration.** All the experiments were run on a single NVIDIA A800 graphics processing unit. Text-to-video inference required 25 min, single-object generation averaged 12 min, and full-scene generation (testing cases averaging 10–20 objects) was completed in 270 min. Compared with existing methods (e.g., text2room [5] requiring 13.5 h), the proposed approach achieved quality improvements with enhanced efficiency.

## Results and Discussion

**Capability analysis and baseline rationale.** Before describing the quantitative and qualitative results, a comprehensive capability comparison between the proposed framework and recent state-of-the-art scene-generation methods is provided (Table 1). Although recent compositional frameworks, such as Set-the-Scene [27], GALA3D [28], and CG3D [29], have successfully enabled object-level editability, they depend heavily on stringent spatial priors, such as explicit 3D bounding boxes or scene graphs provided by the user. In contrast, the proposed approach eliminates reliance on predefined 3D layouts, achieving end-to-end mapping from pure text and trajectories to a decoupled scene representation.

**Table 1:** Capability comparison with recent state-of-the-art scene generation frameworks

Method	Paradigm	Conditioninput	Requires 3D layout	Background extraction	Object editability
DreamFusion [1]	Monolithic	Text	No	*×*	*×*
Text2Room [5]	Monolithic	Text + Trajectory	No	*\checkmark*	*×*
Set-the-Scene [27]	Compositional	Text + Proxy BBoxes	**Yes**	*×*	*\checkmark*
GALA3D [28]	Compositional	Text + Layout Graph	**Yes**	*×*	*\checkmark*
CG3D [29]	Compositional	Text + Scene Graph	**Yes**	*×*	*\checkmark*
**Proposed**	**Compositional**	**Text + Trajectory**	**No**	*\checkmark*	*\checkmark*

Unlike existing compositional methods that rely on heavily constrained spatial priors (e.g., explicit 3D bounding boxes or scene graphs), the proposed framework achieves object-background decoupled generation in an end-to-end manner purely by text and dynamic trajectories. Bold entries indicate the proposed method.*\checkmark*denotes that the corresponding capability is supported;*×*denotes that it is not supported. 3D: Three-dimensional

Because of this fundamental discrepancy in input modalities and problem settings (layout-free *vs* layout-conditioned), directly evaluating these compositional baselines on the continuous background extraction task in this study using standard quantitative metrics (e.g., PSNR and LPIPS) would yield inequitable comparisons. Consequently, the quantitative baselines (Text2Room, Text2NeRF, and DreamScene) in this study were carefully selected to ensure fair evaluation under comparable layout-free input conditions. Furthermore, to rigorously prove the superiority of the proposed object-background decoupling mechanism over monolithic generation without introducing a baseline input bias, a strictly controlled variable analysis was performed in the ablation study.

### Quantitative results

A comprehensive quantitative comparison of the proposed method with all the baselines is presented on standard datasets. As summarized in Table 2, the proposed approach demonstrated superior performance across several key quality metrics.

**Fig. 6:**
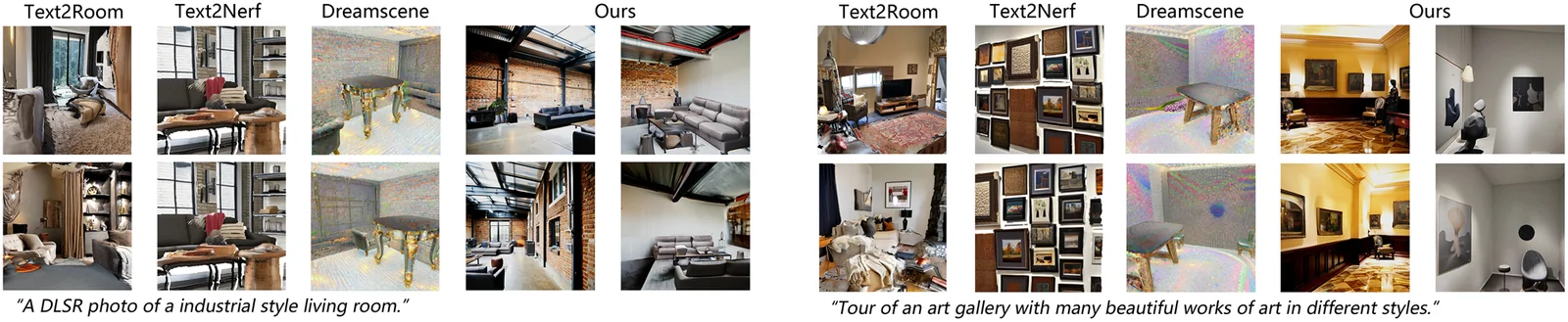
Qualitative comparison on different text prompts. For each prompt, two rendered views are shown from different viewpoints for each method

**Table 2:** Comprehensive evaluation of scene generation quality

Method	PSNR*↑*	LPIPS*↓*	CD (bg/obj)*↓*	EMD (obj)*↓*	Time (h)*↓*
Text2Room	19.5	0.32	0.25/-	-	13.5
Text2NeRF	21.2	0.24	0.18/-	-	7.5
DreamScene	22.1	0.21	0.16/-	0.07	1.0
**Proposed**	**24.2**	**0.20**	**0.011/0.07**	**0.05**	**4.5**

The values represent the average performance of each method on a standard dataset. For baselines that do not support object separation, CD and EMD metrics are evaluated on the entire scene; thus, object-specific metrics are marked as “-”.*↑*indicates that higher is better, and*↓*indicates that lower is better. Bold values denote the results of the proposed method. LPIPS: Learned perceptual image patch similarity; PSNR: Peak signal-to-noise ratio; CD: Chamfer distance; EMD: Earth mover’s distance

The results presented in Table 2 highlight the advantages of the proposed method. In terms of rendering quality, the proposed approach outperformed all the baselines across all the metrics. Notably, for the perceptually critical LPIPS metric, the proposed method achieved a score of 0.20, significantly surpassing the best baseline, DreamScene, which achieved a score of 0.21. This substantial improvement is attributed to the proposed hierarchical generation process, which mitigates feature entanglement and visual artifacts by independently optimizing the background and foreground. Regarding geometric fidelity, the proposed framework is unique in its ability to enable the separate evaluation of individual objects. It achieved a low EMD of 0.05, directly validating its efficacy. In contrast to the monolithic representations used by baselines, the proposed method captures the topology and surface details of objects more accurately, thereby preventing geometric ambiguities and blurring. These results provide strong evidence that the proposed hierarchical paradigm successfully translates architectural innovation into quantifiable gains in terms of both visual realism and geometric precision.

Figure 6 substantiates the quantitative findings in Table 2. In the “industrial-style living room” scene (left), the structural integrity and realism of the proposed method are consistent with its superior PSNR and LPIPS scores, whereas baseline artifacts, such as noise and geometric distortions, directly correlate with their lower perceptual metrics. For the more complex “art gallery” (right), the proposed hierarchical approach preserves instance-level geometry, explaining the superior object-level metrics (CD, EMD) of the proposed method. In contrast, monolithic baseline representations led to object fusion (Text2NeRF) or structural collapse (DreamScene). These results confirmed the superiority of the proposed hierarchical paradigm in delivering both visual fidelity and geometric precision.

To provide a comprehensive perspective on the recent advancements in compositional scene generation, the capabilities of the proposed framework were compared with those of Set-the-Scene [27], GALA3D [28], and CG3D [29] in Table 1. Although these state-of-the-art models similarly achieve object-level editability, they depend heavily on stringent spatial priors, such as explicit 3D bounding boxes or scene graphs provided by the user. In contrast, the proposed approach eliminates reliance on predefined 3D layouts, achieving end-to-end mapping from pure text and trajectories to a decoupled scene representation.

Because of this fundamental discrepancy in the input modalities and problem settings (layout-free *vs* layout-conditioned), directly evaluating these compositional baselines on the continuous multiview background extraction task using quantitative metrics (e.g., PSNR and LPIPS) is computationally intractable and yields inequitable comparisons. Consequently, the quantitative evaluations in Table 2 focus on representative models with comparable layout-free input conditions. Furthermore, to rigorously and quantitatively prove the superiority of the proposed object-background decoupling mechanism over monolithic generation, a strictly controlled variable analysis is provided in subsection “Ablation studies” in which the decoupling module is isolated and evaluated using an identical backbone.

### Ablation studies

#### Robustness under trajectory-conditioned generation

A prominent failure mode in text-to-3D is the loss of scene consistency or semantic fidelity from complex camera-driven viewpoints. To rigorously assess the robustness of the proposed trajectory-conditioned generator, an experiment was designed showing that, regardless of the trajectory complexity, the proposed framework maintains coherent, high-quality outputs–a key requirement for predictable and controllable content creation.

**Experimental design.** Given a set of diverse text prompts, three camera-trajectory variants were synthesized per prompt to examine performance with increasing motion complexity:

1. Linear path camera translates left-to-right or right-to-left along a straight line.
2. Orbital path camera follows a circular (orbiting) trajectory around the region of interest.
3. Complex spline hand-held–style motion with coupled position and rotation changes along a spline curve.

**Evaluation metrics.** Two complementary metrics are reported to quantify the geometry and semantics:

(1) Geometric consistency (MEt3R error *↓*). This study employed MEt3R, an advanced multiview consistency measure that performs test-time reconstruction over the generated frames and quantifies cross-view feature alignment errors; the lower the better. To capture temporal stability over the entire sequence, this study used the standard deviation across consecutive frames as the final score.

(2) Semantic alignment (CLIP score *↑*). The cosine similarity between text and image embeddings from contrastive language-Iimage pre-training (CLIP) was computed for every view, and the mean across frames was reported; a higher value was better.

**Fig. 7:**
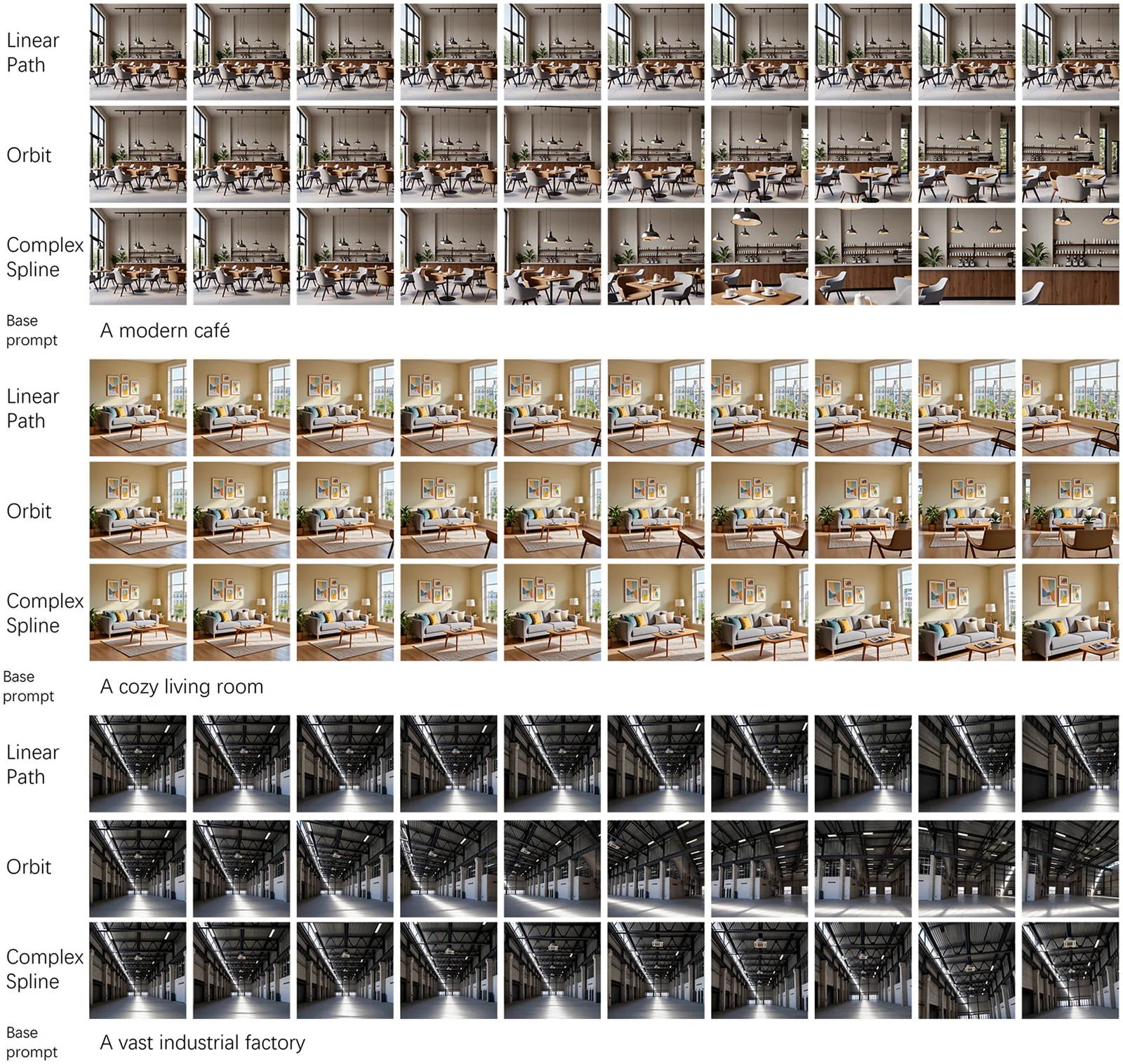
For each prompt, three sequences (linear, orbital, complex spline) are generated. The model maintains geometric coherence and semantic faithfulness across increasing camera-motion complexity

Consistently low MEt3R errors (approximately *0.10*) indicate that the proposed framework preserves geometrically coherent scenes, even under complex navigation. Meanwhile, stable and high CLIP scores (approximately *29*) confirmed that the core semantics remained tightly anchored to the text prompts and were unaffected by camera motion. Taken together, these quantitative findings aligned with the qualitative sequences (Fig. 7) and tabulated statistics Table 3).

**Table 3:** Trajectory-conditioned robustness across prompts

Prompt	Trajectory	**MEt3R***↓*	**CLIP***↑*
A modern café	Linear path	0.03060	27.86
	Orbit	0.08120	26.42
	Complex spline	0.10329	27.27
A cozy living room	Linear path	0.02783	29.86
	Orbit	0.08851	28.78
	Complex spline	0.05015	27.70
A vast industrial factory	Linear path	0.02003	28.89
	Orbit	0.10129	28.54
	Complex spline	0.09591	28.92

Lower MEt3R indicates stronger multiview geometric consistency; higher CLIP reflects better text–image alignment. CLIP: Contrastive language-image pre-training

This analysis provides compelling evidence that the proposed CVAE-based trajectory modulation robustly decouples camera control from scene synthesis, enabling users to specify arbitrary paths without degrading geometric integrity or semantic fidelity.

#### Component-wise contributions

A series of rigorous ablation studies was conducted to isolate and quantify the contributions of each core component within the framework.

A specialized evaluation protocol was devised to assess the efficacy of the scene extraction module. First, a dense sequence of 48 continuous frames was generated to serve as the ground truth. From this sequence, 24 frames were uniformly sampled as sparse input views for reconstruction using the different ablated versions of the proposed model. The remaining 24 unseen frames were then held out and used as a test set for computing evaluation metrics. This process of systematically ablating key modules and measuring the resulting impact on performance enabled precisely attributing the contribution of each component.

The ablation studies listed in Table 4 provide quantitative evidence for the design rationale of the proposed framework and highlight the indispensable role of each core component.

**Fig. 8:**
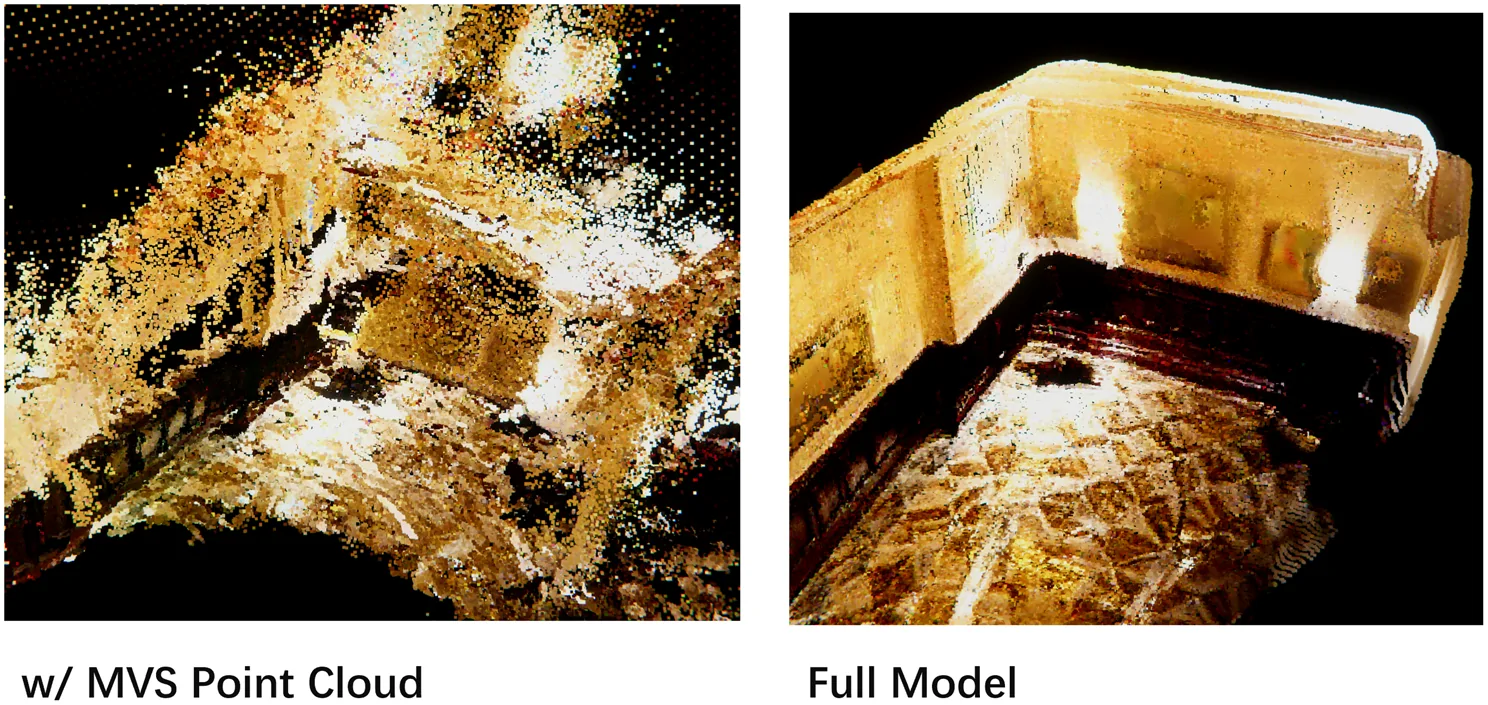
Full, three-dimensional Gaussian splatting-based model (right), which achieves superior geometric integrity and surface smoothness compared with the multiview stereo-based variant (left)

1. SE(3) trajectory control. Removing the explicit SE(3) trajectory control (Variant 2) results in a catastrophic performance collapse, with significant degradation across all the rendering and geometric metrics. This finding proves that trajectory control is not merely a feature for controllability but a fundamental prerequisite for maintaining multiview geometric consistency, without which the reconstruction fails.
2. Hierarchical modeling. Ablating the hierarchical structure (Variant 3) also leads to an across-the-board performance drop. Both geometric quality (F1-score decreases from 0.86 to 0.73) and rendering realism (LPIPS worsens from 0.12 to 0.18) are severely impacted. This confirms the central hypothesis: a monolithic optimization scheme causes severe feature entanglement between the foreground and background, which in turn degrades both geometric precision and visual quality.
3. Comparison with MVS. Finally, compared with a traditional MVS approach (Variant 4), the 3DGS-based optimization pipeline demonstrates greater robustness in handling sparse views. It recovers more precise geometric detail, leading to superior performance on all the metrics, as visualized in Fig. 8.

**Table 4:** Ablation study on the key components of the proposed model

Model variant	**PSNR** *↑*	**SSIM** *↑*	**LPIPS** *↓*	**Precision** *↑*	**Recall** *↑*	**F1-score** *↑*
1. **Full model (proposed)**	**24.2**	**0.90**	**0.12**	**0.88**	**0.85**	**0.86**
2. w/o Ttrajectory control	15.1	0.65	0.41	0.45	0.42	0.43
3. w/o hierarchical modeling	21.5	0.82	0.18	0.75	0.72	0.73
4. w/o MVS point cloud	22.5	0.86	0.15	0.81	0.78	0.79

Standard metrics for rendering quality (PSNR, SSIM, LPIPS) and geometric accuracy (precision, recall, F1-score) are reported.*↑*indicates that higher is better, and*↑*indicates that lower is better. Bold values denote the results of the full proposed model. LPIPS: Learned perceptual image patch similarity; PSNR: Peak signal-to-noise ratio; SSIM: Structural similarity index measure

### Qualitative results

This subsection presents a series of qualitative results to visually corroborate the quantitative evaluations and offers an in-depth analysis of the generative capabilities of the proposed framework. The framework’s performance was assessed across two key dimensions: (1) its ability to synthesize complex scenes with high fidelity and multiview consistency, and (2) the object-level scene editability afforded by its hierarchical representation.

**Fig. 9:**
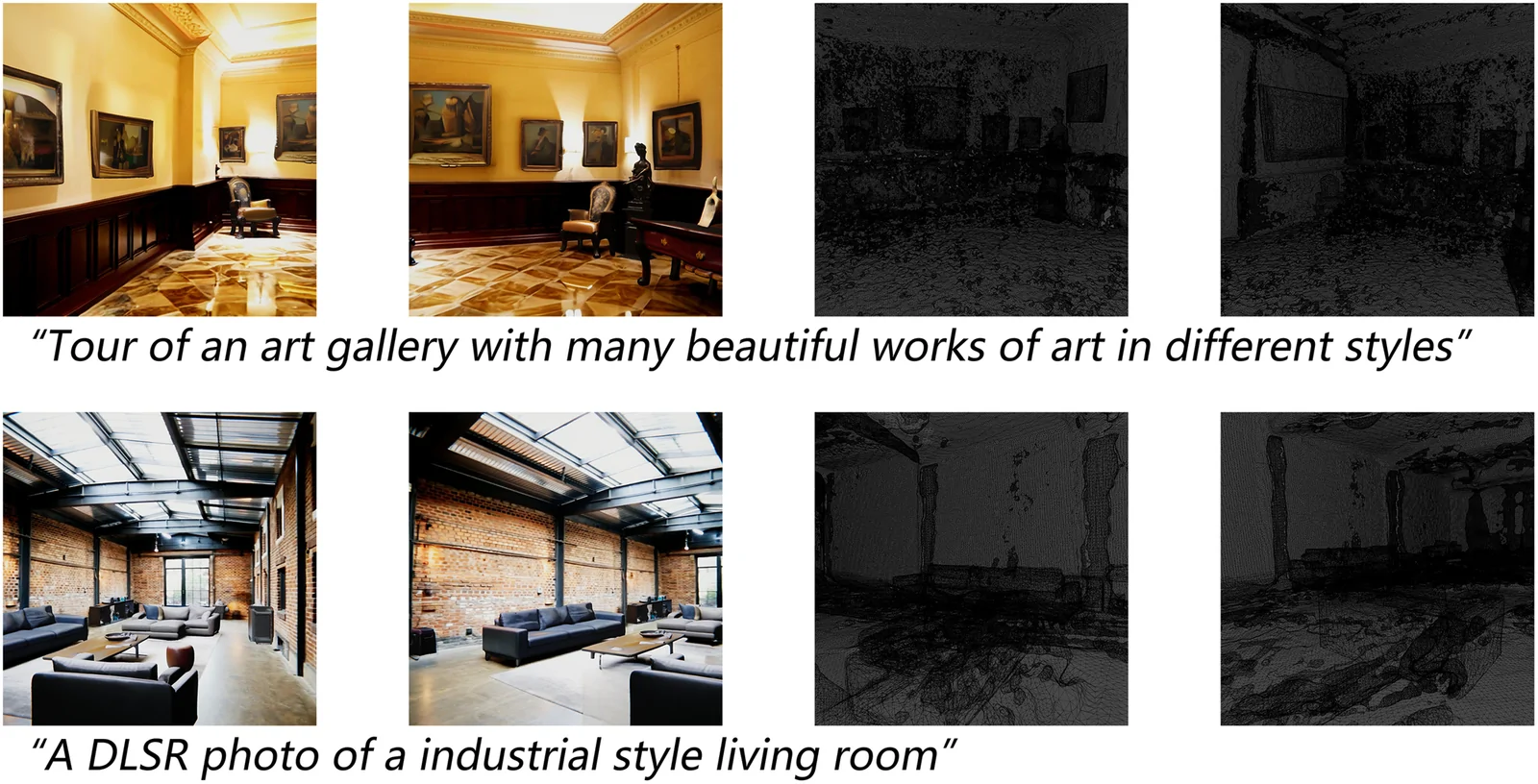
Two scenes generated from text prompts, where for each scene, four discrete views (the last two as wireframe/mesh views) rendered in Unity engine are shown to showcase generation quality and multiview consistency

**Fig. 10:**
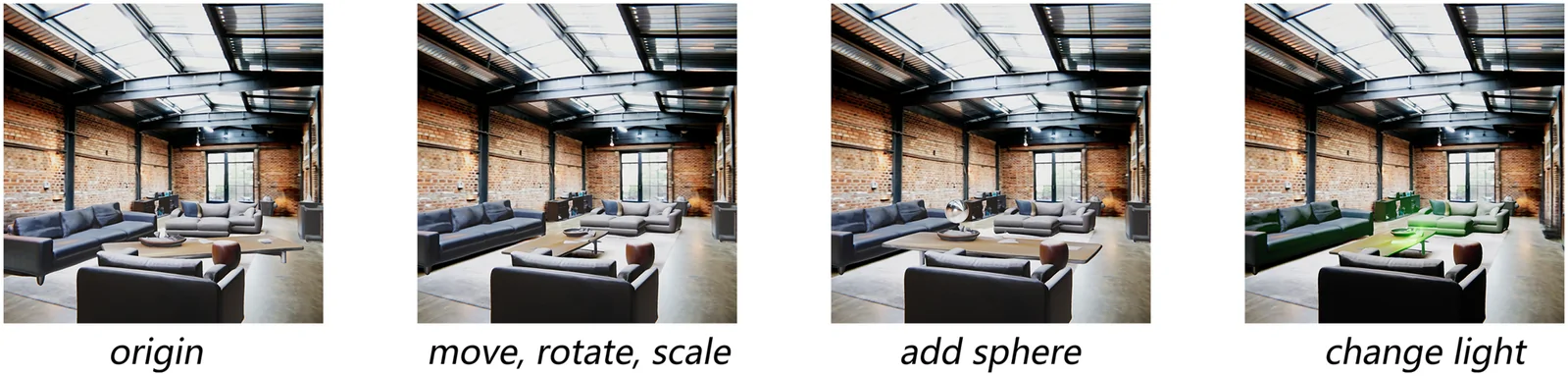
This includes transforming existing objects (moving, scaling, rotating), adding new geometry (a sphere) to the scene, and changing the lighting

**Scene generation quality and multiview consistency**. The visual results shown in Fig. 9 validate the ability of the proposed framework to synthesize complex 3D scenes characterized by high-fidelity rendering and strong internal consistency. In the “art gallery” example, the model demonstrates its capacity to handle heterogeneous materials and complex lighting, rendering scene elements with accurate lighting and shadow effects. Crucially, all the geometric entities maintain strict structural integrity across continuous viewpoint transformations and are free from distortions or interview inconsistencies. Similarly, for an “industrial-style living room” prompt, the proposed model correctly interprets the stylistic semantics and renders the corresponding architectural elements with photorealism. The smooth transitions among the views rendered along a predefined trajectory visually confirmed the high degree of spatial coherence in the 3D representation generated using the proposed method.

**Hierarchical structure and scene editability.** Figure 10 shows the fundamental advantage of the proposed framework: the editability afforded by the hierarchical scene representation. Unlike monolithic approaches, the proposed framework explicitly decouples a scene into distinct background and foreground components, a structural prerequisite for enabling object-level manipulations. To verify downstream compatibility, the hierarchical scene was imported into the Unity engine. As shown, individual foreground instances, such as sofas, can undergo arbitrary rigid-body transformations without compromising the geometric or visual integrity of the scene. Furthermore, the modularity of the framework supports instantiating new geometric entities (e.g., a reflective sphere) that are rendered with physical consistency, as well as modifying global lighting conditions to which all components respond realistically. The ability to perform these edits within a standard engine confirms that the output of the proposed framework transcends static representations. The proposed framework generates structured 3D environments that are not only compatible with existing 3D workflows but are also programmatically editable, providing critical flexibility for downstream interactive applications.

## Conclusions

This study advances text to 3D scene synthesis by introducing a hierarchical architecture that decouples the semantic content, camera trajectory, and geometric representation. A trajectory conditioned diffusion model generates multiview sequences with a consistent structure, whereas cross modal disentanglement and instance tracking isolate objects from backgrounds; 3DGS and an adapted mesh generator provide efficient representations. This combination yields structured scenes with enhanced rendering realism, geometric accuracy, and runtime efficiency compared with recent baselines. Compared with monolithic methods, the proposed approach achieves lower perceptual and geometric errors and can separately evaluate object level geometry, whereas dynamic pruning compresses the background representation without degrading the quality. Ablation analyses verified that both trajectory conditioning and hierarchical modeling are essential to these gains. The decoupled representation maintains coherence across diverse camera paths and supports intuitive object level edits in standard engines, making it applicable to immersive applications, such as virtual reality and digital twins.

### Limitations

This study had several limitations. The framework depends on reliable instance segmentation and tracking; heavy occlusion, rapid motion, or clutter can degrade the mask quality, leading to artifacts during separation and reconstruction. In particular, as a cascaded system, the proposed framework is susceptible to error propagation. Any significant boundary inaccuracies or mis-segmentations produced during the SAM-based segmentation phase can propagate directly to subsequent stages, manifesting as geometric distortions or incompleteness in the final generated 3D meshes. As the background and objects are generated independently, mismatches in lighting or materials may occur when compositing them, particularly under complex illumination. The current evaluation is limited to indoor and synthetic scenes; therefore, the performance in outdoor, highly dynamic, or deformable environments remains uncertain. Although more efficient than some baselines, the pipeline incurs nontrivial computational costs for large or object rich scenes, particularly owing to per object mesh reconstruction.

### Future studies

Future studies should address these limitations and extend the utility of the framework. Incorporating adaptive segmentation and tracking algorithms, potentially augmented with uncertainty estimation, can improve robustness to occlusion and motion. Differentiable physics and relighting models would allow the system to harmonize the appearance of independently generated components, thereby enhancing realism. The joint end to end training of the diffusion, separation, and reconstruction modules can reduce error accumulation and streamline deployment. Expanding the pipeline to support deformable objects and dynamic interactions would broaden its applicability beyond static scenarios. Exploring domain adaptation to handle outdoor scenes, reflective materials, and low light conditions can improve generalization. Finally, coupling hierarchical representations with scene graphs and interactive agents could enable richer semantic editing and simulation, paving the way for practical digital twin and virtual reality applications.

## Data Availability

The datasets generated during and/or analysed during the current study are available from the corresponding author on reasonable request. The primary datasets used in this study, FAIRM and xView, are publicly available resources.

## Abbreviations

3DGS: Three-dimensional Gaussian splatting
CD: Chamfer distance
CLIP: Contrastive language-image pre-training
CVAE: Conditional variational autoencoder
EMD: Earth mover’s distance
KL: Kullback-Leibler
LPIPS: Learned perceptual image patch similarity
NeRF: Neural radiance field
PSNR: Peak signal-to-noise ratio
SAM: Segment anything model
SDF: Signed distance field
SE(3): Three-dimensional special Euclidean group
SSIM: Structural similarity index measure
VCIBA: Visual Computing for Industry, Biomedicine, and Art
VR: Virtual reality
3D: Three-dimensional
2D: Two-dimensional
MVS: Multiview stereo
